# Equivocal expression of emotions in children with Prader-Willi syndrome: what are the consequences for emotional abilities and social adjustment?

**DOI:** 10.1186/s13023-020-1333-9

**Published:** 2020-02-21

**Authors:** Nawelle Famelart, Gwenaelle Diene, Sophie Çabal-Berthoumieu, Mélanie Glattard, Catherine Molinas, Michèle Guidetti, Maithe Tauber

**Affiliations:** 1 CLLE, University of Toulouse, CNRS, Toulouse, France; 2 Université Toulouse Jean Jaurès, Maison de la Recherche, Laboratoire CLLE, 5, allée Antonio Machado, 31058 Toulouse Cedex 9, France; 3 Centre de Référence du Syndrome de Prader-Willi, CHU Toulouse, Toulouse, France; 4 CPTP, University of Toulouse, CNRS, INSERM, Toulouse, France

**Keywords:** Emotion expressions, Prader-Willi syndrome, Children, Social adaptation

## Abstract

**Background:**

People with Prader-Willi Syndrome (PWS) experience great difficulties in social adaptation that could be explained by disturbances in emotional competencies. However, current knowledge about the emotional functioning of people with PWS is incomplete. In particular, despite being the foundation of social adaptation, their emotional expression abilities have never been investigated. In addition, motor and cognitive difficulties - characteristic of PWS - could further impair these abilities.

**Method:**

To explore the expression abilities of children with PWS, twenty-five children with PWS aged 5 to 10 years were assessed for 1) their emotional facial reactions to a funny video-clip and 2) their ability to produce on demand the facial and bodily expressions of joy, anger, fear and sadness. Their productions were compared to those of two groups of children with typical development, matched to PWS children by chronological age and by developmental age. The analyses focused on the proportion of expressive patterns relating to the target emotion and to untargeted emotions in the children’s productions.

**Results:**

The results showed that the facial and bodily emotional expressions of children with PWS were particularly difficult to interpret, involving a pronounced mixture of different emotional patterns. In addition, it was observed that the emotions produced on demand by PWS children were particularly poor and equivocal.

**Conclusions:**

As far as we know, this study is the first to highlight the existence of particularities in the expression of emotions in PWS children. These results shed new light on emotional dysfunction in PWS and consequently on the adaptive abilities of those affected in daily life.

## Introduction

The expression of facial or bodily emotions is an ability that plays a major role in the establishment of interpersonal relationships and thus in social adaptation. In some pathologies, many difficulties in social adjustment are observed, which may be related to disturbance in the manifestation and sharing of emotions.

Prader-Willi syndrome (PWS) is a rare genetic disease (birth incidence rate of 1: 20,000 to 1: 25,000) related to the loss of expression of some paternal inherited genes on chromosome 15 region q11–13. This leads to significant dysfunction of the hypothalamic-pituitary hormonal and neurohormonal system. PWS is a complex neurodevelopmental disorder characterized by severe neonatal hypotonia with deficits of sucking and swallowing and anorexic behavior that may induce failure to thrive, and which subsequently change to excessive weight gain and obesity with hyperphagia and deficits of satiety [1]. The phenotype also comprises learning difficulties and many psychological dysfunctions. In terms of cognitive abilities, people with PWS display a mild or moderate intellectual disability (average IQ of 60–70), memory, executive and perceptive dysfunctions [1–5]. They exhibit a language and a motor delay [6–9]. In terms of social abilities, people with PWS show social adjustment difficulties and many behavioral disorders [1, 7, 10–12].

The loss of the expression of the inherited paternal genes of the chromosome region 15q11-q13 can be caused by a paternal chromosomal deletion of various length (deletion subtype) or by the inheritance of two maternal chromosomes 15 (maternal disomy – mUPD subtype). In very rare cases, mutations, epimutations of the imprinting center or translocations involving this region are observed [13]. Many studies have reported phenotypic differences according to the genetic subtype between deletion and disomy. PWS people with a deletion have more severe physical impairment (i.e. facial dysmorphia, hypotonia, obesity) [1, 14, 15], stronger behavioral disorders (i.e. externalized disorders and attention-deficit disorders with hyperactivity) and a higher degree of emotional lability [5, 15]. They display more language and communication difficulties [5, 16], impaired memory span and lack of inhibition [17, 18]. The phenotype associated with mUPD is characterized by a less severe hypotonia and obesity [1, 14, 15]. Individuals display better oral and verbal skills [5, 16] but more visual-perceptive deficits, sluggishness in cognitive execution [17, 18], and with more autistic features and severe psychiatric issues [1, 19, 20].

Although socio-emotional maladjustment and behavioral disorders in people with PWS are part of the phenotype, there is currently little knowledge of the mechanism of these disorders. Concerning emotional aspects, the literature describes a symptomatology such as tantrums, emotional lability, impulsive behaviour, lack of empathy and of emotional regulation, anxiety and difficulties of social adaptation [5, 7, 11, 19, 21, 22], suggesting disturbances in social and emotional competencies (i.e. ability to use emotions daily [23];). However, few studies have been conducted to precisely characterize the emotional functioning of people with PWS, hindering appropriate clinical care. The few studies conducted on this subject report difficulties in the recognition and comprehension of basic emotions. In particular, individuals with PWS make on average 10 to 20% more errors in identifying and assigning emotions than the typical population, even when matched for developmental age [5, 21]. Certain information processing particularities among people with PWS suggest that these particularities could be partly responsible for these shortcomings. Individuals with PWS take very little information into account to judge a situation. They focus on details that are mostly irrelevant and have great difficulty in figuring a global representation of the situation [10, 24, 25]. Regarding face analysis, they tend to neglect the eye area whereas it is the part of the face that contains the most information about expressions (particularly for the distinction of negative emotions). Conversely, they tend to focus on the central and bottom part of the face (i.e. the nose). This is particularly observed in subjects with a disomy [26, 27]. This particularity is likely to compromise their capacity for emotional recognition and thus to place them at a disadvantage in everyday situations (i.e. adaptation).

**Fig. 1:**
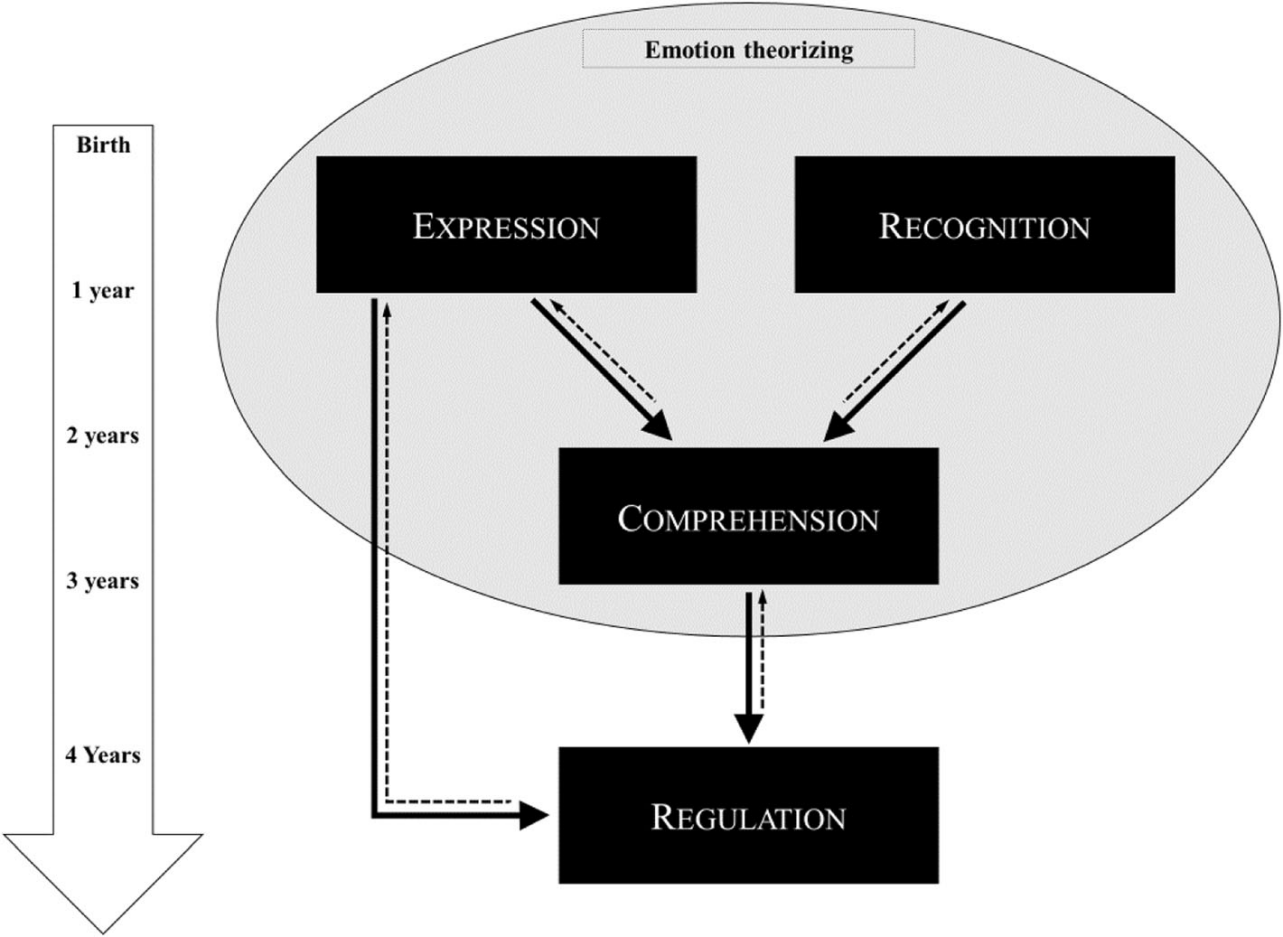
Developmental model of emotion competencies

To date, the expression and emotional regulation skills in PWS have never been investigated. As a result, we do not have a complete vision of the emotional development of people with PWS. In typical development, there is a hierarchy in the emergence of emotional competences during childhood [28–30]. The expression and recognition of emotional skills constitute basic developmental abilities that emerge very early, during the first months of life. They contribute greatly to the development of the comprehension of emotions. Expression, recognition and comprehension skills allow the individual to conceive of emotion as a concept (an ability that we propose to call “emotion theorizing” and that refers to a process first pointed out by Thommen, Dumas, Erskine & Reymond, [31]), necessary for the regulation of emotions and its repercussions on general adaptation (see Fig. 1). Emotional expression is at the foundation of the establishment of interpersonal relationships and therefore of social adaptation. It corresponds to the first mode of communication of infants with their entourage [29, 32, 33]. It also reflects the capacity for body control, which is the basis of some emotional regulation strategies [34].

Emotional expressions - whether facial, vocal or bodily - are the result of muscle mobilization controlled by the cortical (pyramidal circuit) and subcortical (extrapyramidal circuit) motor system [35]. These two systems are independent but interact with each other: the cortical motor system is involved in voluntary expressions, while the subcortical system is more involved in spontaneous facial expressions. In terms of expressive skills, one can then distinguish spontaneous emotional reactions from deliberate emotional productions. These two abilities are highly involved in social adaptation, whether for the communication of emotion or for the control of one’s own expression. Beyond neuro-motor abilities, the expression of emotions and its control also require cognitive (knowledge of pattern expressions) and executive abilities (inhibition, attentional) [34, 36].

Our current knowledge about the emotional functioning of people with PWS and even more about its development during childhood is incomplete. In particular, the emotional expression abilities (facial, bodily) have never been investigated. The aim of this study was to explore the expression abilities in children with PWS through a task of spontaneous emotional reactions to a video clip and a task of voluntary productions of emotional expressions. In view of the multiple disorders related to this syndrome (and in particular at the neuro-motor, cognitive, communicational and social levels), we suggest that children suffering from PWS exhibit particularities in the expression of their emotions that contribute to their difficulties in emotional recognition, comprehension, regulation skills and social adjustment.

## Method

### Participants

The study population was composed of 25 children with PWS aged 5;5 to 10;5 years (*M* = 7;6 [years; months]; *SD* = 1;6; 14 girls). The average IQ was 75.7 (range = 44 to 103). Thus, the average intellectual developmental age of these children was 5;7 years (*SD* = 1;5; range = 3;2 to 9;2 years). Eleven children had a deletion, twelve had a mUPD and the genetic subtype was unknown in two children. Details about sex, ages and IQ by PWS genetic subtype group are presented in Table 1. The recruitment of children was carried out through the PWS French National Reference Centre.

**Table 1:** Descriptive characteristics for the two PWS genetic subtype groups

	All PWS (N = 25)	PWS-DEL (*N* = 11)	PWS-UPD (*N* = 12)	Group equivalence test * Student t-test
CA				
Mean (SD)	7;6 (1;6)	7;4 (1;5)	7;9 (1;9)	t_(21)_ = 0.529; ns
Range	5;5–10;5	5;5–9;7	5;9–10;4	
DA				
Mean (SD)	5;7 (1;5)	5;9 (1;7)	5;5 (1;4)	t_(21)_ = 0.517; ns
Range	3;2–9;2	3;2–9;1	4;3–9;1	
IQ				
Mean (SD)	75.7 (17.1)	78.4 (14.7)	72.9 (19.5)	t_(21)_ = 0.751; ns
Range	44–103	52–94	44–103	

Legend. Means, ranges and SDs of chronological age (CA), intellectual developmental age (DA) and full-scale IQ (IQ) for the two PWS genetic subtype groups and results of group equivalence tests. *ns*: not significant, age: [years; months], PWS-DEL: PWS with deletion, PWS-UPD: PWS with mUPD

Fifty children with typical development (TD) also participated in the study, divided into two groups. The first group consisted of 25 children matched to PWS children by sex and chronological age (TD-CA: *M* = 7;6 years; *SD* = 1;5; range = 5;2 to 10;10 years; 14 girls). The second group consisted of 25 children matched to PWS children by sex and intellectual developmental age (TD-DA: *M* = 5;7 years; *SD* = 1;4; range = 3;0 to 8;4; 14 girls). None of the TD children had any academic or learning delay.

Only voluntary children with informed parental consent participated in the study. In line with the latest Declaration of Helsinki, all the children and parents were fully informed of the nature and characteristics of the study.

### Materials and methods analysis

#### Spontaneous emotional reactions task (‘EMOrea task’)

A funny video clip (from Famelart & Guidetti [37]) that was likely to induce the emotion of joy in children was used. The clip was shown on a laptop computer and the children’s facial reactions were recorded via the webcam.

Facial reactions were analyzed with FaceReader [38], a software for the automatic analysis of emotional facial expressions. It is based on the FACS system [39], which breaks down facial expressions into individual components of movement, called Action Units (AUs). The expression of each emotion corresponds to a specific combination of AUs (i.e. an AU pattern). For example, the combination of AUs in the Joy pattern is AUs^1^ [12] + [6] + [25]: activation of the Zygomatic involving raising of the corners of the lips (open or closed mouth) and creasing (or not) of the corners of the eyes.

FaceReader analyzes the intensity of each emotion (e.g. joy, anger, sadness, fear, disgust and surprise) in facial expressions, and attributes a value between 0 and 1: 0 means that the emotion is not visible in the facial expression, 1 means that the emotion is fully detectable.

#### Voluntary production of emotional expressions task (‘EMOmim task’)

The second task was created to assess the child’s ability to voluntarily express the emotions of joy, fear, sadness and anger. The child stands in front of a tripod camera and the whole body is filmed.

A coding grid was created to analyze facial expressions on the one hand, and bodily expressions on the other hand. Facial expressions were coded using a combination of the MAX/AFFEX system [40, 41] and the FACS system [39]. Both systems are based on the same coding method, i.e. facial expressions are broken down into AUs. The MAX / AFFEX system proposes a simplified division which emphasizes the main AUs engaged in emotional expressions, taking into account the context of expression as well as inter-individual variability. The adaptation applied in this study consisted in slightly specifying the MAX/AFFEX system by including more detailed descriptors from the FACS system (for more information on these two systems, see Sullivan and Lewis [42]). Bodily expressions were coded with the BEEOS system [43] which is based on the same coding method as FACS and MAX / AFFEX. The Elan software [44], an assistance application for the annotation and transcription of video, was used to annotate all the AUs (facial and bodily AUs) expressed by the child for each of the emotion conditions. Combinations of AUs produced in each emotion condition were then matched with a theoretical pattern by emotion, expressed as a proportion between 0 and 1.

All the video material was analyzed by a principal coder who was naïve to the experiment. To ensure the reliability of the coding grid, 24% of the video material was randomly assigned to a second coder who was also naive to the experiment. The level of agreement between the two coders was then assessed. The reliability can be considered good and adequate for both facial (Cohen’s *k* = 0.63) and bodily expressions (Cohen’s *k* = 0.68). For more information about the interpretation of the FACS system reliability, see for example Sayette et al. [45].

#### Social adaptation skills

The Socio-Affective Profile (PSA: Profil Socio-Affectif; Dumas, Lafrenière, & Capuano [46]) is a French questionnaire designed to evaluate the social adaptation capacities of children aged 2 to 6. It is completed by a parent or educator. It contains 80 items presented in the form of statements about the child’s behavior regarding the expression of his/her affectivity and the characteristics of his/her social interactions with other children and with adults. For each statement, the adult indicates the frequency of behavior observed in the child along the following continuum: 1. Never; 2. Rarely; 3. Occasionally; 4. Regularly; 5. Often; 6. Always.

The PSA provides a score for the child on four global scales: social competence, internalized problems, externalized problems and finally the general adaptation index. The raw score for each scale is converted into a standardized score which can range from 30 to 70 points. The central average is 50 and 80% of the normal distribution is between 38 to 62 points; beyond these values, the scores correspond to atypical profiles.

The French version used here has very good psychometric properties since it shows a good internal consistency for the four global scales (Cronbach’s alpha varying from .79 to .92). In addition, PSA has good inter-rater reliability (from .70 to .91) as well as good test-retest reliability (from .70 to .87) and temporal stability (beyond 6 months; .59 to .76). Finally, the PSA has a very good convergent validity with the CBCL (Child Behavior Checklist; Edelbrock & Achenbach [47]).

#### Procedure

Each child was individually interviewed in a quiet room at home. The EMOrea Task was first proposed: children were asked to watch the video clip on the laptop computer without any other instructions. The experimenter stood behind the child to avoid the child trying to engage in discussion. Secondly, the EMOmim Task was proposed. Children, standing in front of the experimenter and the camera, were asked to mimic expressions of joy, anger, fear and sadness (i.e. *“Show me how you express that you’re happy / angry / sad / afraid”)*. During this phase, the parents completed the PSA.

## Results

To test the hypothesis that children with PWS present specificities in the expression of emotions, analyses were conducted by comparing the four groups of children: two groups of children with typical development matched by chronological age (TD-CA) and by developmental age (TD-DA); two groups of PWS children, one with the mUPD subtype (PWS-UPD) and the other with the deletion subtype (PWS-DEL).^2^ The analyses focused on the proportion of expressive patterns relating to the target emotion and to untargeted emotions in children’s productions. This approach was chosen to characterize the expression profile of PWS children, that is, if this is an overall weakness in the mobilization of facial expressions (e.g. poor facial expressions) or if this involves inappropriate movements (such as the presence of movements from expressive pattern relating to the untargeted emotion).

### EMOrea task: comparisons of the expression patterns in spontaneous emotional reactions between PWS and TD children

A one-way ANOVA (group factor: TD-CA vs. TD-DA vs. PWS-DEL vs. PWS-UPD) was performed on each emotion expression pattern (i.e. joy, anger, fear, sadness, surprise and disgust).

**Fig. 2:**
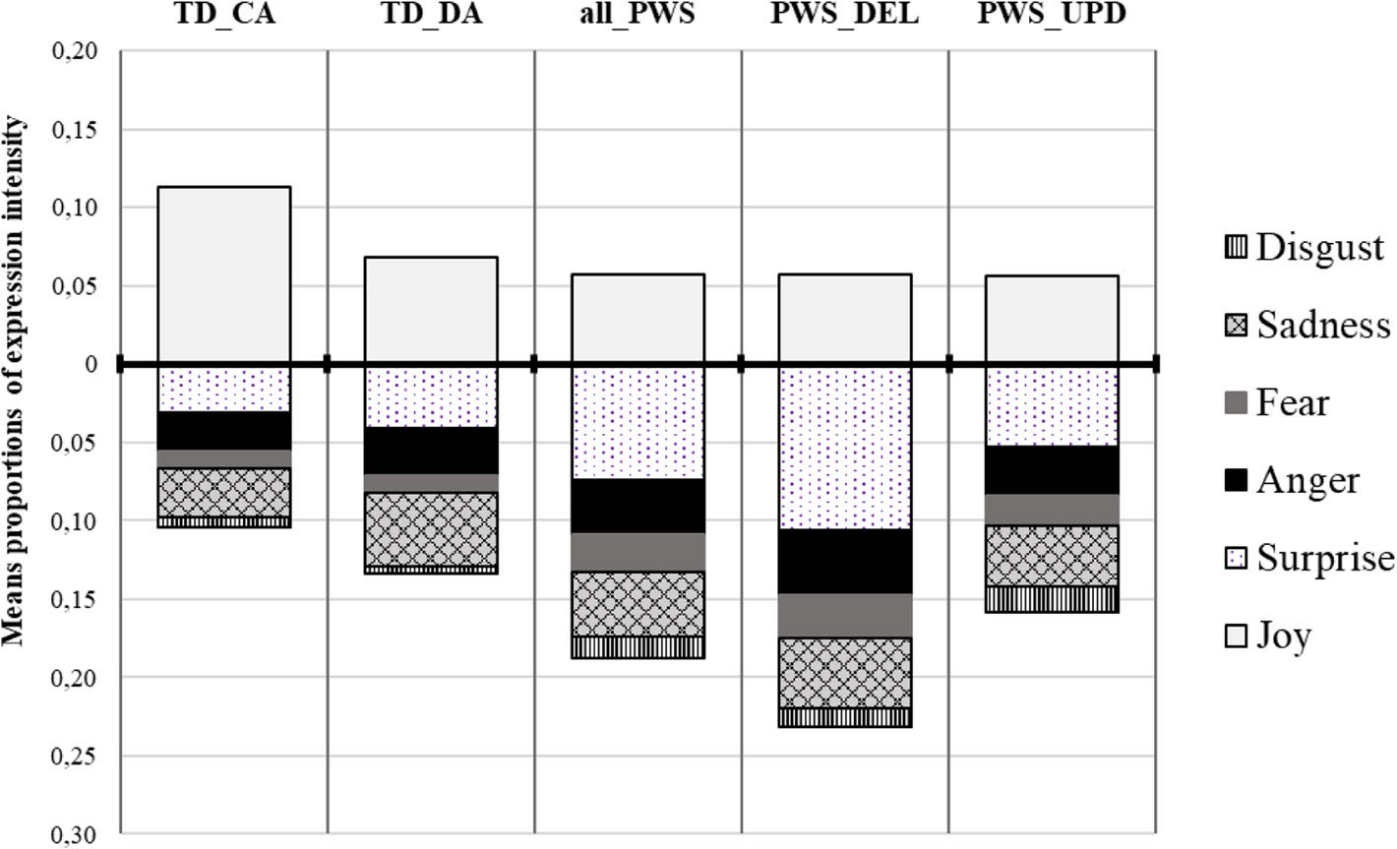
Mean proportion of AUs from each emotion pattern in the children’s facial reactions (EMOrea task). Legend. Upper part of the figure: target emotion; lower part: untargeted emotions

Table 2 presents the means and SDs of the proportion of AUs from each emotion pattern in the children’s facial reactions by group, and the summary results of the one-way ANOVA analyses. Figure 2 illustrates the results. The upper part of the figure refers to the target emotion (i.e. joy), while the lower part refers to the untargeted emotions.

**Table 2:** Proportion of AUs in the children’s facial reactions (EMOrea task)

Emotion pattern	All-PWS (*N* = 25)	PWS-DEL (*N* = 11)	PWS-UPD (*N* = 12)	TD-CA (*N* = 25)	TD-DA (*N* = 25)	Group effect (One-way ANOVA)	Post-hoc comparison (Tukey correction)
Joy^a^	0.057 (0.048)	0.057 (0.046)	0.056 (0.054)	0.113 (0.130)	0.068 (0.082)	*ns*	
Surprise	0.074 (0.110)	0.106 (0.157)	0.053 (0.047)	0.031 (0.072)	0.041 (0.091)	*ns*	
Anger	0.035 (0.028)	0.041 (0.029)	0.031 (0.029)	0.025 (0.023)	0.030 (0.038)	*ns*	
Fear	0.024 (0.018)	0.028 (0.017)	0.019 (0.020)	0.011 (0.012)	0.011 (0.009)	*p* = .003	DEL > UPD / DA / CA
Sadness	0.041 (0.030)	0.028 (0.017)	0.019 (0.020)	0.031 (0.025)	0.047 (0.055)	*ns*	
Disgust	0.014 (0.010)	0.012 (0.008)	0.016 (0.012)	0.006 (0.010)	0.005 (0.004)	*p* < .001	UPD > DEL / DA / CA

Legend. Means (SDs) proportion of AUs from each emotion pattern in the children’s facial reactions by group, and the summary results of One-way ANOVA analyses. ^a^Target emotion; *ns* not significant

Analyses showed a significant group effect on the proportion of AUs from the patterns of fear (*F*_(3,69)_ = 5.186; *p* = .003) and disgust (*F*_(3,69)_ = 6.404; *p* < .001). The post hoc comparisons with Tukey correction showed that the proportion of AUs from the Fear pattern was significantly higher in the PWS-DEL group than in the TD-DA group (*p* = .005) and the TD-CA group (*p* = .005). No significant differences were observed between the PWS-DEL and UPD groups. Conversely, the proportion of AUs from the ‘disgust’ pattern was significantly higher in the PWS-UPD group than in the TD-DA group (*p* = .001) and the TD-CA group (*p* = .009), whereas no significant differences were observed with the PWS-DEL group.

The four groups did not differ significantly in the proportion of AUs from the patterns of joy, surprise, sadness and anger.

### EMOmim task: comparisons of expression patterns in voluntary productions of emotion between PWS and TD children

As the frequency distribution of the dependent variable (i.e. Proportion of AUs) was not normal, we used a Generalised Linear Model (GLM) based on a Binomial distribution with a logit link function adapted to proportion data. We selected ‘Group’ as the only factor (TD-CA vs. TD-DA vs. PWS-DEL vs. PWS-mUPD).

A GLM analysis was performed for each emotion pattern (i.e. joy, anger, fear and sadness), in each expression modality (i.e. facial, bodily) and in each mimic condition (i.e. happiness, anger, fear, sadness).

#### Happiness condition

**Fig. 3:**
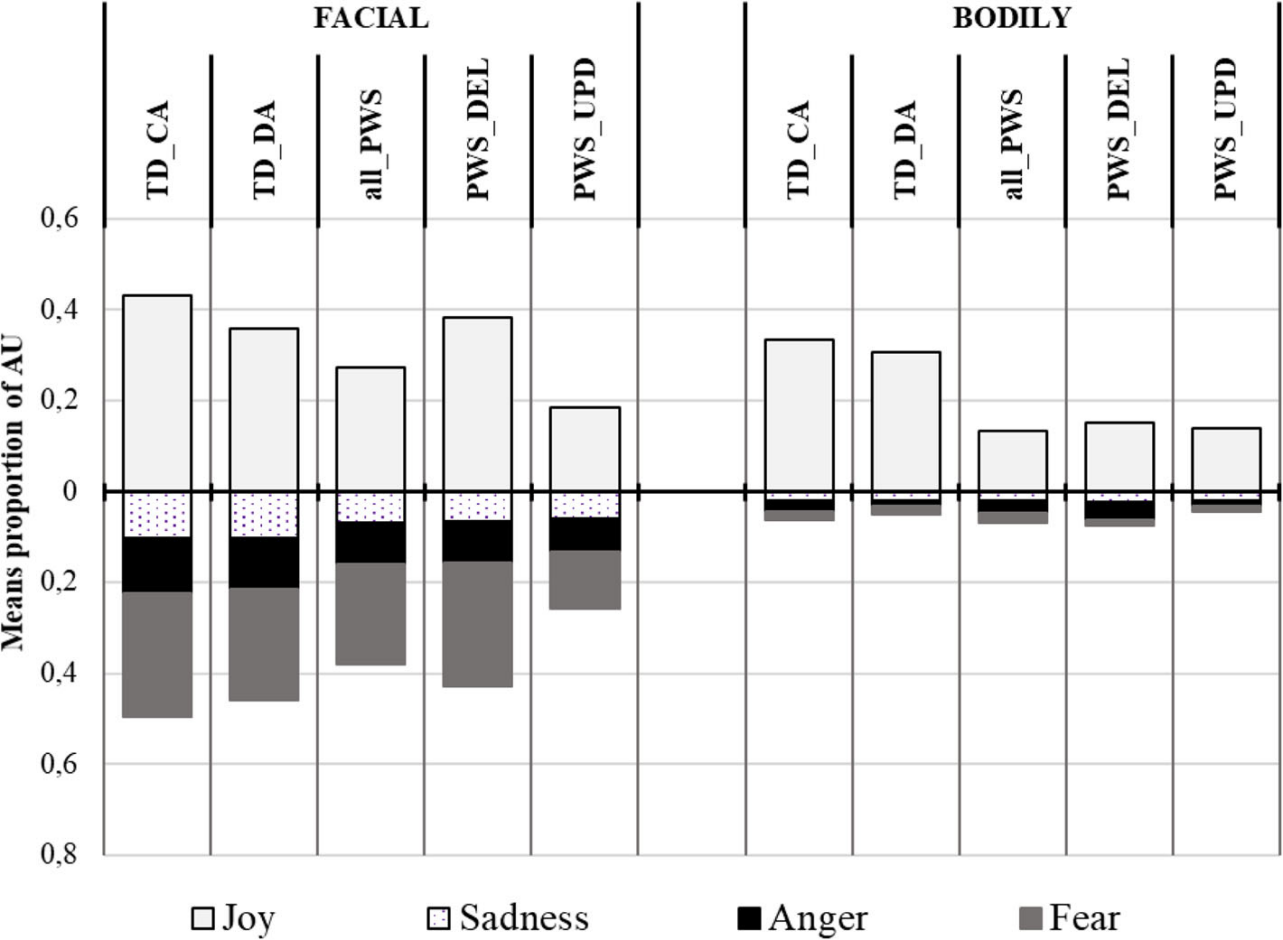
Mean proportion of AUs in the happiness condition (EMOmim task). Legend. Proportion of AUs from each emotion pattern in the children’s facial and bodily productions. Upper part of the figure: target emotion; lower part: untargeted emotions

Table 3 presents the means and SDs of the proportion of AUs from each emotion pattern in facial and bodily productions for the four groups, and the summary results of the GLM analyses. Figure 3 illustrates the results. The upper part of the figure refers to the target emotion (i.e. joy), while the lower part refers to the untargeted emotions.

**Table 3:** Proportion of AUs in the happiness condition (EMOmim task)

Modality	Emotion pattern	All-PWS (*N* = 25)	PWS-DEL (*N* = 11)	PWS-UPD (*N* = 12)	TD-CA (*N* = 25)	TD-DA (*N* = 25)	GLM analyses	
							Group effect *(Wald Chi-Square test)*	Pairwise comparisons *(Wald test)*
FACIAL	Joy*	0.272 (0.182)	0.382 (0.188)	0.184 (0.134)	0.432 (0.180)	0.358 (0.186)	*p* = .008	UPD < DEL / DA / CA
	Sadness	0.069 (0.073)	0.064 (0.074)	0.060 (0.073)	0.103 (0.087)	0.101 (0.066)	*ns*	
	Anger	0.091 (0.100)	0.091 (0.096)	0.071 (0.096)	0.120 (0.099)	0.113 (0.084)	*ns*	
	Fear	0.220 (0.185)	0.273 (0.187)	0.125 (0.103)	0.273 (0.165)	0.243 (0.177)	*p* = .064	UPD < DEL / DA / CA
BODILY	Joy*	0.133 (0.193)	0.150 (0.230)	0.140 (0.170)	0.333 (0.137)	0.307 (0.093)	*p* = .043	UPD / DEL < DA / CA
	Sadness	0.020 (0.070)	0.023 (0.075)	0.020 (0.073)	0.020 (0.070)	0.020 (0.070)	*ns*	
	Anger	0.029 (0.059)	0.039 (0.067)	0.011 (0.041)	0.023 (0.053)	0.011 (0.040)	*ns*	
	Fear	0.020 (0.055)	0.015 (0.050)	0.013 (0.048)	0.020 (0.055)	0.020 (0.057)	*ns*	

Legend. Means (SDs) proportion of AUs from each emotional expression pattern in the children’s facial and bodily productions and summary results of GLM analyses. *Target emotion; *ns* not significant

Analyses showed a significant group effect on the proportion of facial AUs from the Joy pattern (Wald Chi-Square test; *χ*^*2*^_(*N* = 72, 3)_ = 11.896; *p* = .008). The PWS-UPD group mobilized significantly fewer AUs from the Joy pattern than the TD-CA group (Wald test: *z* = 3.216, *p* = .001), the TD-DA group (*z* = 2.372, *p* = .018) and the PWS-DEL group (*z* = 2.332, *p* = .020). These three groups did not differ from one another.

Moreover, the proportion of facial AUs relating to the untargeted emotion patterns (i.e. anger and sadness) was statistically similar between the four groups. Nevertheless, we observed differences from the Fear pattern (*χ*^*2*^_(*N* = 72, 3)_ = 7.277; *p* = .064, tendency). The PWS-UPD group showed fewer AUs from the Fear pattern than the TD-CA group (*z* = 3.416, *p* = .016), the TD-DA group (*z* = 1.996, *p* = .046) and the PWS-DEL group (*z* = 2.140, *p* = .032), whereas the other three groups did not differ from one another.

In this condition, analyses also showed a significant group effect on the proportion of bodily AUs from the Joy pattern (*χ*^*2*^_(N = 72, 3)_ = 8.109; *p* = .043). The PWS-UPD group significantly fewer AUs than the TD-CA group (*z* = 2.093, *p* = .036) and a trend to fewer AUs than the TD-DA group (*z* = 1.839, *p* = .066). The trend in the PWS-DEL group was also towards fewer AUs than in the TD-CA group (*z* = 1.893, *p* = .058). The proportion of bodily AUs from the untargeted emotion patterns (i.e. anger, fear and sadness) was statistically similar between the four groups.

#### Anger condition

**Fig. 4:**
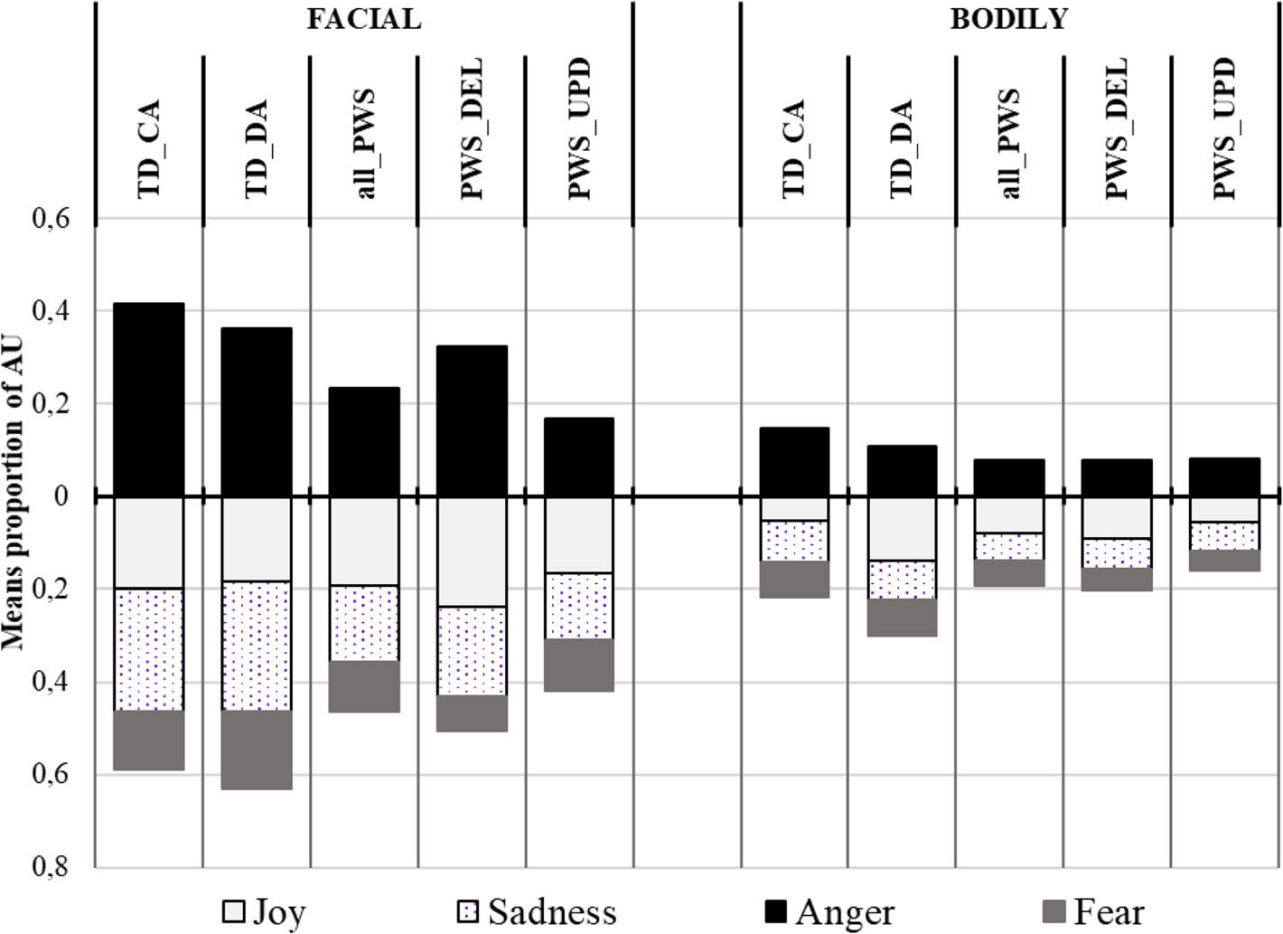
Mean proportion of AUs in the anger condition (EMOmim task). Legend. Proportion of AUs from each emotion pattern in the children’s facial and bodily productions. Upper part of the figure: target emotion; lower part: untargeted emotion

Table 4 presents the means and SDs of the proportion of AUs from each emotion pattern in facial and bodily productions for the four groups, and the summary results of the GLM analyses. Figure 4 illustrates the results. The upper part of the figure refers to the target emotion (i.e. anger), while the lower part refers to the untargeted emotions.

**Table 4:** Proportion of AUs in the anger condition (EMOmim task)

Modality	Emotion pattern	All-PWS (*N* = 25)	PWS-DEL (*N* = 11)	PWS-UPD (*N* = 12)	TD-CA (*N* = 25)	TD-DA (*N* = 25)	GLM analyses	
							Group effect *(Wald Chi-Square test)*	Pairwise comparisons *(Wald test)*
FACIAL	Joy	0.192 (0.122)	0.236 (0.120)	0.166 (0.116)	0.200 (0.100)	0.184 (0.116)	*ns*	
	Sadness	0.166 (0.129)	0.194 (0.131)	0.143 (0.121)	0.263 (0.129)	0.280 (0.136)	*p* = .054	UPD < DA / CA
	Anger*	0.234 (0.184)	0.324 (0.203)	0.167 (0.134)	0.417 (0.164)	0.363 (0.179)	*p* < .001	UPD < DEL / DA / CA
	Fear	0.107 (0.135)	0.075 (0.115)	0.112 (0.148)	0.126 (0.155)	0.167 (0.138)	*ns*	
BODILY	Joy	0.080 (0.147)	0.090 (0.157)	0.057 (0.130)	0.053 (0.123)	0.140 (0.193)	*ns*	
	Sadness	0.060 (0.110)	0.068 (0.118)	0.063 (0.113)	0.090 (0.143)	0.083 (0.140)	*ns*	
	Anger*	0.080 (0.101)	0.079 (0.117)	0.083 (0.096)	0.149 (0.127)	0.107 (0.153)	*ns*	
	Fear	0.053 (0.080)	0.045 (0.078)	0.042 (0.075)	0.073 (0.085)	0.077 (0.085)	*ns*	

Legend. Means (SDs) proportion of AUs from each emotional expression pattern in the children’s facial and bodily productions and summary results of GLM analyses. *Target emotion; *ns* not significant

In this condition, analyses showed a significant group effect on the proportion of facial AUs from the Anger pattern (*χ*^*2*^_(*N* = 72, 3)_ = 17.635; *p* < .001). The PWS-UPD group mobilized significantly fewer AUs than the TD-CA group (*z* = 3.858, *p* < .001), the TD-DA group (*z* = 3.138, *p* = .002) and the PWS-DEL group (*z* = 2.304, *p* = .021). These three groups did not differ from one another.

Moreover, the proportion of facial AUs from untargeted emotion patterns (i.e. joy and fear) was statistically similar between the four groups. Nevertheless, we observed differences between groups from the Sadness pattern (*χ*^*2*^_(*N* = 72, 3)_ = 7.661; *p* = .054, tendency). The PWS-UPD group showed significantly fewer AUs from the Sadness pattern than the TD-CA group (*z* = 2.137, *p* = .033) and the TD-DA group (*z* = 2.376, *p* = .018). The proportions in the PWS-DEL group were similar to those of the PWS-UPD group and to both the TD-CA and TD-DA groups.

Analyses also showed no group effect on the proportion of bodily AUs from the Anger pattern (*χ*^*2*^_(N = 72, 3)_ = 3.943; *ns*). The proportion was similar between the PWS-UPD group, the PWS-DEL group, the TD-CA group and the TD-DA group. The proportion of bodily AUs from the untargeted emotion patterns (i.e. joy, fear and sadness) was also statistically similar between the four groups.

#### Sadness condition

**Fig. 5:**
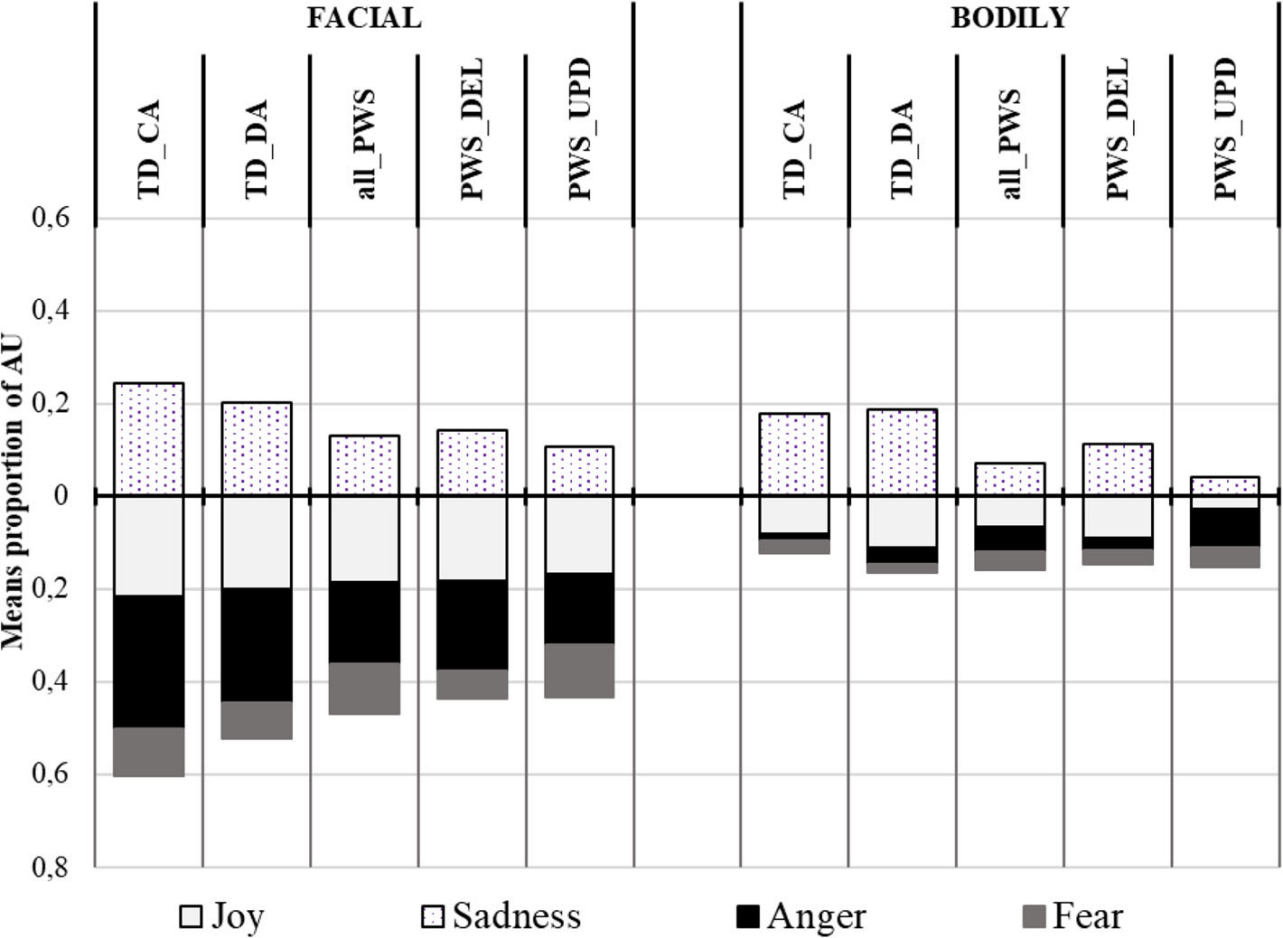
Mean proportion of AUs in the sadness condition (EMOmim task). Legend. Proportion of AUs from each emotion pattern in the children’s facial and bodily productions. Upper part of the figure: target emotion; lower part: untargeted emotion

Table 5 presents the means and SDs of the proportion of AUs from each emotion pattern in facial and bodily productions for the four groups, and the summary results of the GLM analyses. Figure 5 illustrates the results. The upper part of the figure refers to the target emotion (i.e. sadness), while the lower part refers to the untargeted emotions.

**Table 5:** Proportion of AUs in the sadness condition (EMOmim task)

Modality	Emotion pattern	All-PWS (*N* = 25)	PWS-DEL (*N* = 11)	PWS-UPD (*N* = 12)	TD-CA (*N* = 25)	TD-DA (*N* = 25)	GLM analyses	
							Group effect *(Wald Chi-Square test)*	Pairwise comparisons *(Wald test)*
FACIAL	Joy	0.184 (0.128)	0.182 (0.108)	0.166 (0.144)	0.216 (0.114)	0.200 (0.118)	*ns*	
	Sadness*	0.131 (0.091)	0.143 (0.064)	0.107 (0.107)	0.246 (0.127)	0.203 (0.119)	*p* = .030	UPD / DEL < DA / CA
	Anger	0.177 (0.133)	0.194 (0.096)	0.154 (0.166)	0.286 (0.170)	0.244 (0.149)	*p* = .090	UPD < CA
	Fear	0.107 (0.173)	0.060 (0.112)	0.112 (0.205)	0.100 (0.145)	0.077 (0.163)	*ns*	
BODILY	Joy	0.067 (0.137)	0.090 (0.157)	0.027 (0.097)	0.080 (0.147)	0.110 (0.160)	*ns*	
	Sadness*	0.070 (0.115)	0.113 (0.130)	0.043 (0.098)	0.180 (0.235)	0.188 (0.185)	*p* = .045	UPD < DA / CA
	Anger	0.051 (0.109)	0.026 (0.086)	0.083 (0.129)	0.017 (0.047)	0.036 (0.076)	*p* = .091	UPD < CA
	Fear	0.040 (0.087)	0.030 (0.067)	0.042 (0.103)	0.027 (0.062)	0.020 (0.057)	*ns*	

Legend. Means (SDs) proportion of AUs from each emotional expression pattern in the children’s facial and bodily productions and summary results of GLM analyses. *Target emotion; *ns*: not significant

Analyses showed a significant group effect on the proportion of facial AUs relating to sadness (*χ*^*2*^_(*N* = 72, 3)_ = 8.937; *p* = .030). The PWS-UPD group mobilized significantly fewer AUs from the Sadness pattern than the TD-CA group (*z* = 2.534, *p* = .011) and showed a trend towards fewer than the TD-DA group (*z* = 1.864, *p* = .062). The proportion in the PWS-DEL similar to that of the PWS-UPD group and the TD-DA group, but with a trend towards fewer AUs than the TD-CA group (*z* = 1.811, *p* = .070).

The proportion of facial AUs relating to untargeted emotions (i.e. joy and fear) was statistically similar between the four groups. Nevertheless, we observed differences from the Anger pattern (*χ*^*2*^_(*N* = 72, 3)_ = 6.490; *p* = .090, tendency). The PWS-UPD group showed fewer AUs from the Anger pattern than the TD-CA group (*m* = 0.29, *sd* = 0.17; *z* = 2.265, *p* = .024), whereas the other three groups did not differ from one another.

In this condition, analyses also showed a significant group effect on the proportion of bodily AUs from the Sadness pattern (*χ*^*2*^_(*N* = 72, 3)_ = 8.068; *p* = .045). The PWS-UPD group displayed significantly fewer AUs than the TD-CA group (*z* = 2.109, *p* = .035) and the TD-DA group (*z* = 2.173, *p* = .030). The proportion in the PWS-DEL group was similar to those of the PWS-UPD group and the TD-CA and TD-DA groups. The proportion of bodily AUs from the untargeted emotion patterns (i.e. fear and joy) was statistically similar between the four groups. Nevertheless, we observed differences from the Anger pattern (*χ*^*2*^_(N = 72, 3)_ = 6.455; *p* = .091, tendency). The PWS-UPD group showed significantly more bodily AUs from the Anger pattern than the TD-CA group (*z* = 2.347, *p* = .019), whereas the other three groups did not differ from one another.

#### Fear condition

**Fig. 6:**
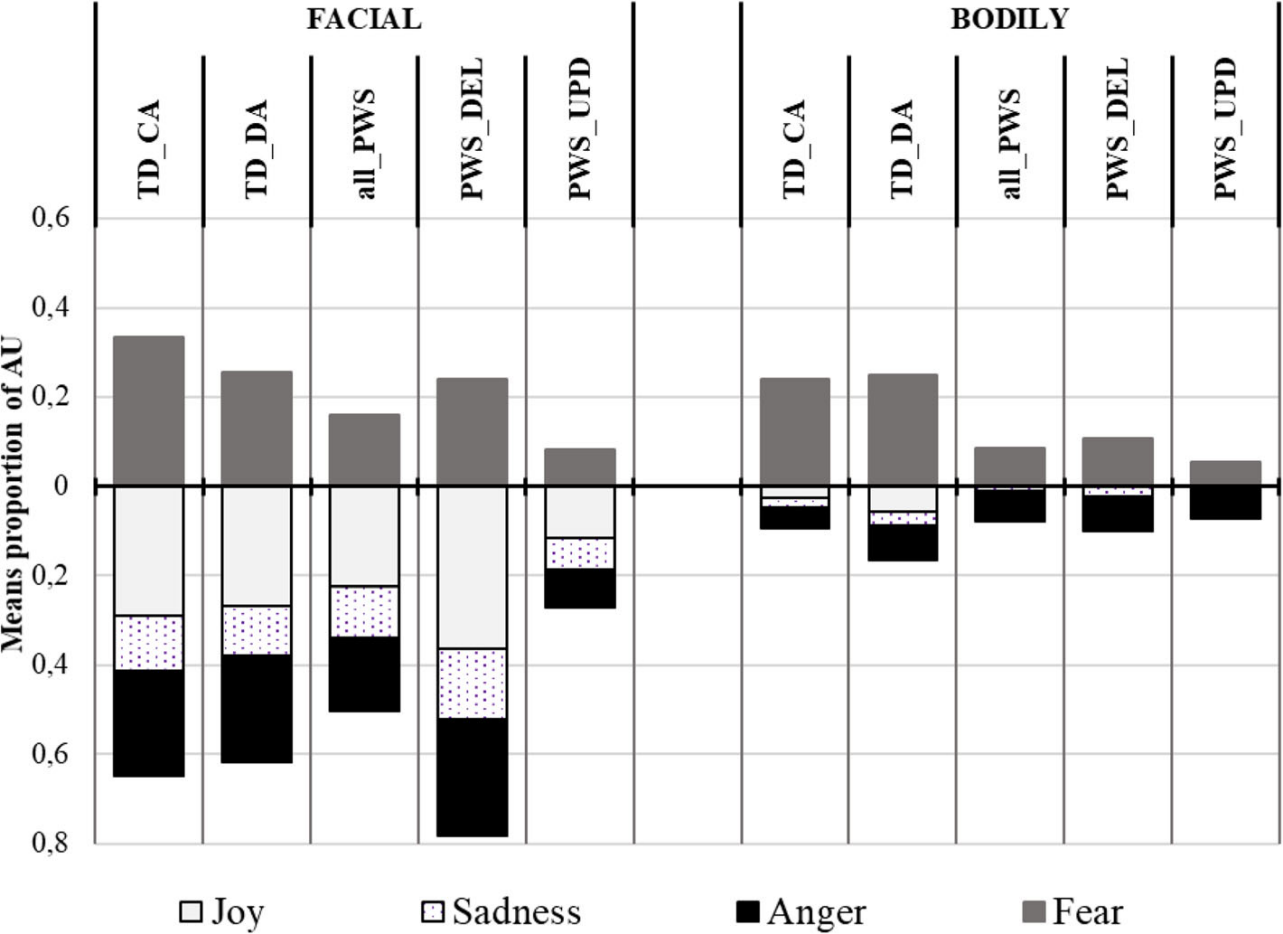
Mean proportion of AUs in the fear condition (EMOmim task). Legend. Proportion of AUs from each emotion pattern in the children’s facial and bodily productions. Upper part of the figure: target emotion; lower part: untargeted emotion

Table 6 presents the means and SDs of the proportion of AUs from each emotion pattern in facial and bodily productions for the four groups, and the summary results of the GLM analyses. Figure 6 illustrates the results. The upper part of the figure refers to the target emotion (i.e. fear), while the lower part refers to the untargeted emotions.

**Table 6:** Proportion of AUs in the fear condition (EMOmim task)

Modality	Emotion pattern	All-PWS (*N* = 25)	PWS-DEL (*N* = 11)	PWS-UPD (*N* = 12)	TD-CA (*N* = 25)	TD-DA (*N* = 25)	GLM analyses	
							Group effect *(Wald Chi-Square test)*	Pairwise comparisons *(Wald test)*
FACIAL	Joy	0.224 (0.202)	0.364 (0.134)	0.116 (0.144)	0.288 (0.142)	0.266 (0.128)	*p* = .030	UPD < DEL / DA / CA
	Sadness	0.114 (0.083)	0.156 (0.074)	0.071 (0.107)	0.126 (0.063)	0.113 (0.084)	*ns*	
	Anger	0.166 (0.141)	0.260 (0.096)	0.083 (0.166)	0.234 (0.116)	0.239 (0.144)	*p* = .006	UPD < DEL / DA / CA
	Fear*	0.16 (0.19)	0.242 (0.133)	0.083 (0.205)	0.333 (0.16)	0.257 (0.203)	*p* < .001	UPD < DEL / DA / CA
BODILY	Joy	0 (0)	0 (0)	0 (0)	0.027 (0.093)	0.057 (0.127)	*ns*	
	Sadness	0.010 (0.050)	0.023 (0.075)	0 (0)	0.020 (0.070)	0.030 (0.085)	*ns*	
	Anger	0.069 (0.110)	0.079 (0.099)	0.071 (0.129)	0.046 (0.090)	0.077 (0.084)	*ns*	
	Fear*	0.087 (0.098)	0.107 (0.112)	0.055 (0.082)	0.240 (0.167)	0.250 (0.177)	*p* < .001	UPD / DEL < DA / CA

Legend. Means (SDs) proportion of AUs from each emotional expression pattern in the children’s facial and bodily productions and summary results of GLM analyses. *Target emotion; *ns* not significant

In this condition, analyses showed a significant group effect on the proportion of facial AUs from the Fear pattern (*χ*^*2*^_(*N* = 72, 3)_ = 18.562; *p* < .001). The PWS-UPD group mobilized significantly fewer AUs from the Fear pattern than the TD-CA group (*z* = 3.704, *p* < .001), the TD-DA group (*z* = 2.860, *p* = .004) and the PWS-DEL group (*z* = 2.448, *p* = .014). These three groups did not differ from one another.

The proportion of facial AUs from the pattern of sadness was statistically similar between the four groups. Nevertheless, we observed significant groups differences from the Anger pattern (*χ*^*2*^_(N = 72, 3)_ = 12.376; *p* = .006) and the Joy pattern (*χ*^*2*^_(*N* = 72, 3)_ = 10.903; *p* = .012). The PWS-UPD group showed significantly fewer AUs relating to the Anger pattern than the TD-CA group (*z* = 2.801, *p* = .005), the TD-DA group (*z* = 2.843, *p* = .004) and the PWS-DEL group (*z* = 2.858, *p* = .004), whereas these three groups did not differ from one another. The same effect was observed concerning the proportion of AUs from the Joy pattern: the PWS-UPD group showed a significantly lower proportion than the TD-CA group (*z* = 2.498, *p* = .012), the TD-DA group (*z* = 2.241, *p* = .025) and the PWS-DEL group (*z* = 2.988, *p* = .003). These three groups did not differ from one another.

Analyses also showed a significant group effect on the proportion of bodily AUs from the Fear pattern (*χ*^*2*^_(N = 72, 3)_ = 19.937; *p* < .001). The PWS-UPD group showed significantly fewer AUs than the TD-CA group (*z* = 3.062, *p* = .002) and the TD-DA group (*z* = 3.158, *p* = .002). The proportion in the PWS-DEL group was similar to that of the PWS-UPD group but significantly lower than in the TD-CA group (*z* = 2.209, *p* = .027) and in the TD-DA group (*z* = 2.328, *p* = .020). The proportion of bodily AUs from the untargeted emotion patterns (i.e. joy, anger and sadness) was statistically similar between the four groups.

### Relation between emotional expression abilities and social adaptation skills (PSA)

#### Comparison of social adaptation skills between PWS and TD children

A one-way ANOVA (group factor: TD-CA vs. TD-DA vs. PWS-DEL vs. PWS-UPD) was performed on each PSA scale (i.e. social competence, internalized problems, externalized problems, and general adaptation).

Analyses showed a significant group effect on the General Adaptation Scale (*F*_(3,69)_ = 15.62; *p* < .001), the Social Skills Scale (*F*_(3,69)_ = 29.26; *p* < .001) and the Internalized Problems scale (*F*_(3,69)_ = 11.40; *p* < .001).

The *post-hoc* comparisons with Tukey correction showed that scores of General Adaptation were significantly weaker in the PWS-DEL group (*m* = 43.2, *sd* = 5.0) than in the TD-DA group (*m* = 51.1, *sd* = 5.2; *p* = .002) and the TD-CA group (*m* = 54.0, *sd* = 6.0; *p* < .001). Scores were also significantly weaker in the PWS-UPD group (*m* = 42.9, *sd* = 6.5) than in both the TD-DA (*p* < .001) and TD-CA groups (*p* < .001). No significant differences were observed between the PWS-DEL and UPD groups.

The same trend was observed in the scores of Social Skills. Scores were significantly lower in the PWS-DEL group (*m* = 49.9, *sd* = 5.3) than in the TD-DA group (*m* = 53.9, *sd* = 5.9; *p* < .001) and the TD-CA group (*m* = 56.3, *sd* = 4.8; *p* < .001). Scores were also significantly weaker in the PWS-UPD group (*m* = 45.2, *sd* = 5.2) than in both the TD-DA (*p* < .001) and TD-CA groups (*p* < .001). No significant differences were observed between the PWS-DEL and UPD groups.

On the Internalized Problems Scale, scores in the PWS-DEL group (*m* = 44.1, *sd* = 4.3) were significantly weaker than in the TD-CA group (*m* = 50.9, *sd* = 6.8; *p* = .02) but did not differ from the TD-DA group (*m* = 48.2, *sd* = 5.7; *ns*). Scores in the PWS-UPD group (*m* = 41.0, *sd* = 7.5) were significantly weaker than in both the TD-DA (*p* < .008) and TD-CA groups (*p* < .001). No significant differences were observed between the PWS-DEL and UPD groups.

On the Externalized Problems Scale, no significant difference was observed between the scores of the PWS-DEL group (*m* = 42.5, *sd* = 3.3), the PWS-UPD group (*m* = 45.7, *sd* = 5.1), the TD-CA group (*m* = 46.6, *sd* = 7.1) and the TD-DA group (*m* = 44.8, *sd* = 5.2).

#### Correlations between emotional expression abilities and social adaptation skills

Spearman correlation (with Holm correction) analyses were performed between the four PSA scales, the global proportion of emotional expressions used in the EMOrea task and in the EMOmim task. The correlation matrix is presented in Table 7.

**Table 7:** Matrix correlation between the PSA scales, EMOrea and EMOmim (global proportion of AUs)

	1	2	3	4	5
1. PSA-General Adaptation	–				
2. PSA-Social Skills	.93 ***	–			
3. PSA-Internalied Problems	.77 ***	.59 ***	–		
4. PSA-Externalized Problems	.60 ***	.42 ***	.32 **	–	
5. EMOrea (global)	−.11	−.11	−.01	−.14	–
6. EMOmim (global)	.44 ***	.49 ***	.42 ***	−.03	.01

Legend. Matrix *r* results of Spearman correlation (with Holm correction). Correlation significant at: *: *p* < .05; **: *p* < .01; ***: *p* < .001

Results indicated that the global performance in EMOmim was significantly correlated with three scales of the PSA. We observed a positive relation between the global proportion of AUs mobilized in EMOmim and the scores in general adaptation, social skills and internalized problems. No correlation was observed with spontaneous emotional reactions in the EMOrea task.

#### Correlations between IQ, emotional expression abilities and social adaptation skills in PWS children

No significant correlation (Spearman correlation with Holm correction) was observed between the IQ and the global performance in EMOmim and EMOrea. The results followed the same trend between the IQ and three scales of the PSA (general adaptation, social skills and internalized problems). However, we observed a positive relation between the IQ level and the scores in externalized problems of the PSA (*r* = .52, *p* = .007).

## Discussion

The purpose of this study was to explore the emotional expression abilities of children with PWS, which has never been investigated until now even though it is an early ability that plays a crucial role in child development. In view of the multiple neuro-motor, cognitive, communicational and social disorders associated with PWS, we assumed that children with PWS would display particularities in the expression of emotions (whether spontaneous or voluntary) that play a role in their difficulties in emotional skills and social adjustment. Interestingly, the results reveal that the facial and bodily emotional expressions of children with PWS were particularly equivocal, and in many cases the mimics were poor.

The analysis of spontaneous reactions to a funny video clip (EMOrea task) showed a higher proportion of AUs from the patterns of disgust (mUPD subtype) and fear (Deletion subtype) in children’s laughter expressions. When analysed in depth, it turns out that the smiles of children with PWS are more equivocal because of a stronger raising of the upper lip (which corresponds to AU 9 from the ‘disgust’ pattern) or a stronger stretching outward of the lip corners that reduces the raising (which corresponds to AU 20 from the Fear pattern).

The analyses of the voluntary productions (EMOmim task) showed that these equivocal expressions are particularly prominent in PWS children with mUPD. For each emotional expression condition (i.e. anger, sadness, joy, and fear), the proportion of AUs relative to the target emotion was significantly lower than among the TD children and the PWS-DEL children, while the proportion of AUs from other untargeted emotions remained similar across all groups. In other words, children with mUPD display much more equivocal emotional productions, with a majority of AUs related to untargeted emotions. In addition, children with mUPD have a lower overall rate of AUs, making their facial (but not bodily) expressions particularly poor.

These results therefore highlight specificities in the spontaneous expression of emotions in children with PWS and in voluntary productions mainly in the mUPD subtype. Although the majority of the children’s expressions are understood by their close entourage and especially when they are contextualized, their weak and equivocal expression can create significant difficulties during the first interactions between the infant and his/her parents. This greatly complicates the relationships between the parents and their baby and therefore the establishment of the attachment bond. These results make it possible to characterize the subtlety of the modes of interaction of PWS individuals, which opens up new perspectives concerning early care. Importantly, the same difficulties are observed with peers at early ages that are also crucial for socialization.

Expressive abilities can refer, among other things, to neuro-motor abilities, which are particularly impaired in PWS [48, 49]. These deficits seem to be responsible for peculiarities such as equivocal spontaneous expressiovns, which refer to the subcortical system. On the other hand, the difficulties in voluntary expressions (cortical system) are only observed in children with mUPD, who display poor expression. This raises the question of how/why these two circuits are impacted in this syndrome and whether the genetic profile can be involved. These aspects deserve to be explored more specifically in future studies. Veltman et al. [14] suggested that motor coordination difficulties (fine motor skills) are more pronounced in people with mUPD. This agrees with our results: the ambiguity in voluntary productions could be the result of a difficulty in coordinating the mobilization of the AUs necessary to express an emotion. In addition, the muscle mass deficit is higher in people with mUPD [50]. These considerations should be taken into account during early psychomotor care.

The existence of difficulties in voluntary expression in the UPD subtype could also be related to the high salience of autistic disorders among this group. Indeed, the peculiarities in voluntary expression could be linked to a lack of interest in interaction, and less willingness to communicate [14, 20, 21, 51]. This aspect could reinforce the difficulties and explain the poor facial expressions. Imitative capacities deserve to be investigated and trained in these children, which would lead to therapeutic methods adapted according to the patient’s profile. Significantly impaired oxytocin (OXT) neurones have been demonstrated in people with PWS [52] and various PWS mice models [53, 54] obtained by inactivating some imprinted genes of the paternal inherited chromosomal region. Intranasal OXT administrations have been used in neonates/infants with PWS and improved oral and social skills after 7 days [55]. In addition, facial expression and motor coordination were improved in these infants and remained after 3 years. A complementary approach using early OXT treatment and reinforcing imitative capacities may be useful in this syndrome and other neurodevelopmental disorders.

The study of the scores of the four global scales of the PSA tells us that children with PWS are different from TD children and have lower adaptive skills, and more specifically lower social abilities. According to the literature, internalized problems are particularly present in PWS, especially in the UPD subtype [11, 21, 56]. This element is also linked to the poor expression of emotions in these children. In fact, the correlation analyses highlight a positive relationship between voluntary expression capacity and social adaptation. Furthermore, emotional expression abilities of PWS children were weaker than TD children matched by the intellectual developmental age. We also did not find correlation between expression skills and the IQ. Thus, difficulties in emotional expression abilities seem to be a specificity of the PWS, and not directly depend on the cognitive impairment (that seems more related to the externalized behavioural disorders). These results precise the socio-emotional profile and the bases of the social maladjustment in the PWS.

The results show a relatively clear emotional expression profile trend and in line with the PWS literature. However, we must be cautious about generalizing these results in view of the small sample and the high inter-individual variability. In addition, although both genetic subtype groups have statistically equivalent IQs in this study, cognitive impairment remains an important consideration in explaining the results. To overcome these limitations, future studies will need to be conducted with larger samples. Furthermore, to better understand the development of emotional expression skills in the context of PWS, these skills should also be explored in a longitudinal approach with younger children (e.g. 0–2 years old, 3–5 years old), as well as with older children (e.g. 10–16 years old) and adults.

## Conclusion

Voluntary expression skills involve the capacity for body control, which is crucial for certain emotional regulation strategies and also contributes to social adaptation. It is understandable then that peculiarities in the emotional expression (itself related to disorders of body control, motor coordination, but also of communication) could play a major role in difficulties of emotional regulation and social adaptation. This reinforces the idea that emotional expression is the foundation of interpersonal relationships. Other studies should be conducted to analyze their involvement in other emotional skills such as recognition and understanding of emotions. Overall, this study suggests that there is interest in promoting and supporting the development of the expressive capacities of these children. Offering an early care program would improve the children’s relationship with their parents, which is fundamental to their development.

## Data Availability

The datasets used and/or analyzed during the current study are available from the corresponding author on reasonable request.

## Abbreviations

AU: Action Unit
CA: Chronological Age
DA: Developmental Age
DEL: Deletion
FACS: Facial Action Coding System
MAX / AFFEX: MAXimally Discriminative AFFEct Coding System
mUPD: maternal UniParental Disomy
PSA: Socio-Affective Profile (french version: Profil Socio-Affectif)
PWS: Prader-Willi Syndrome
TD: Typical Development

## Footnotes

1 AU 12: Lip Corner Puller (Zygomatic Major); AU 6: Cheek Raiser (Orbicularis oculi, pars orbitalis); AU 25: Lips Part (Depressor Labii, Relaxation of Mentalis, Orbicularis Oris).

2 The two PWS children with an unknown genetic subtype was included only in the all-PWS group vs TD group comparisons.
